# Unraveling the Puzzle: A Case of Intricate Neurological Presentation Attributable to Hypomagnesemia

**DOI:** 10.7759/cureus.69273

**Published:** 2024-09-12

**Authors:** Syed H Hussain, Mohammed Zaidi, Mariam Zaidi, Guy M Grabau

**Affiliations:** 1 College of Osteopathic Medicine, Kansas City University, Joplin, USA; 2 Department of Internal Medicine, Freeman Hospital West, Joplin, USA; 3 College of Osteopathic Medicine, Arkansas College of Osteopathic Medicine, Fort Smith, USA

**Keywords:** bidirectional nystagmus, electrolyte deficiencies, covid-19, neurological symptoms due to hypomagnesemia, seizures, hypomagnesemia

## Abstract

Hypomagnesemia can occasionally present with severe neurological deficiencies, and it is usually attributed to an underlying renal and/or gastrointestinal pathology. Rarely, patients may present with neurological symptoms in the absence of an obvious cause. Our case highlights the importance of considering hypomagnesemia as a primary cause of those presenting with severe neurological deficits in an intensive care unit setting, as well as the significance of conducting a thorough social and medical history on these patients to elucidate their underlying causes. We discuss the case of a 48-year-old Caucasian male who acutely presented with seizures, tremors, visual hallucinations, diplopia, personality changes, and ataxia with recurring severely low magnesium (0.4 mg/dL) at times in the absence of renal, gastrointestinal, hormonal, infectious, or autoimmune pathology.

## Introduction

Magnesium is an element that is essential to many biochemical functions of the human body. The cation serves as a cofactor for RNA enzymes, DNA repair processes, and over 300 different enzymes involved in metabolic pathways such as glycolysis, Krebs cycle, and ion transport across cell membranes. Furthermore, magnesium plays a crucial role in regulating adenosine triphosphate production [1,2].

Only 1% of the total body's magnesium is present in extracellular fluid [3]. In a healthy adult, the normal values of magnesium within the body are in the range of 1.46-2.48 mg/dL. A magnesium level of below 1.46 mg/dL classifies as hypomagnesemia. However, symptoms do not generally arise until serum magnesium levels fall below 1.2 mg/dL [4]. After ruling out parathyroid hormone abnormalities, there are two primary mechanisms behind magnesium loss: renal and gastrointestinal. Renal losses are due to a disruption in magnesium reabsorption within the proximal and/or distal tubule. This can be secondary to medications such as thiazide and loop diuretics or due to congenital defects as seen in Gitelman's syndrome and Bartter's syndrome [5]. Gastrointestinal losses are seen primarily due to deficiencies in the lower gastrointestinal tract with the most common cause being chronic diarrhea. Diarrhea is thought to contribute to greater magnesium loss than vomiting as the lower gastrointestinal secretions can be as high as 15 mEq/L compared to 1 mEq/L in upper gastrointestinal tract secretions [6].

According to more recent literature, another possible gastrointestinal cause of hypomagnesemia is chronic proton pump inhibitor use. In a large cohort study consisting of 11,490 patients who were admitted to a single-facility intensive care unit, it was found that those taking omeprazole concurrently with a diuretic were found to have a 0.028 mg/dL lower serum magnesium level compared to those not on a proton pump inhibitor. Additionally, it was found that the prevalence of hypomagnesemia (cutoff being less than 1.6 mg/dL) was significantly higher in patients on both omeprazole and diuretics compared to those who were only on diuretics (15.6% versus 11%) [7]. In patients admitted to the intensive care unit, the incidence of hypomagnesemia can be between 60% and 65% alluding to the importance for clinicians to recognize and promptly treat such high-acuity patients [6]. We hereby put forth a case of a 48-year-old Caucasian male who presents with intricate neurological symptoms secondary to hypomagnesemia.

## Case presentation

A 48-year-old Caucasian male with a documented past medical history of hypertension, gout, gastroesophageal reflux disease, remote alcohol use disorder, and pulmonary embolism that occurred three months ago presented to the emergency department with a chin laceration due to a recent fall. Over the past month, he had progressively worsening dizziness, vertigo, ataxia, weight loss, and personality changes without an overt underlying cause. Suddenly, in the emergency department, he began having a generalized tonic-clonic seizure lasting 1-2 minutes. He was subsequently intubated for airway protection due to post-seizure obtundation. He was transferred to the intensive care unit and was sedated with propofol and midazolam, and he was also started on seizure prophylaxis with levetiracetam 1,500 mg twice daily. Laboratory work revealed hypomagnesemia of 0.4 mg/dL. He was given an initial 2 g of intravenous magnesium sulfate given over 60 minutes. Repeat magnesium one hour later showed a value of 1 mg/dL. He was then given an additional 4 g of magnesium sulfate intravenously over a 12-hour period. He was also supplemented for concomitant electrolyte abnormalities, namely, hypokalemia and hypocalcemia. Of note, his parathyroid level was 218.2 pg/mL, which corroborates the physiological principle that when calcium and/or magnesium is low, it stimulates parathyroid release.

The next morning, his magnesium levels were 1.6 mg/dL. The patient's physical examination also revealed fine tremors in all extremities, which worsened with stimulation and passive movement. A continuous 24-hour electroencephalogram (EEG) showed no epileptiform activity. Computed tomography (CT) of the brain without contrast, CT angiography of the head and neck, and magnetic resonance imaging (MRI) did not show any acute vascular pathology or intracranial processes. Two days after admission, the patient was successfully weaned off sedation and extubated. His magnesium levels were now 2.2 mg/dL. The patient was still confused and endorsed diplopia with new-onset visual hallucinations. His fine tremors had disappeared. Magnesium levels dropped to 1.8 mg/dL, and he was once again loaded with an additional 2 g of intravenous magnesium. Due to serum magnesium only showing 1% of the body's true magnesium level [3], it was clear that his persistently low values indicated a total body loss of magnesium. We decided to start him on a daily oral supplementation of magnesium chloride 64 mg two times daily with the intention of restoring his intracellular storage and preventing further neurological issues.

The patient's remote history of alcohol use disorder along with the presenting triad of ophthalmoplegia, confusion, and ataxia raised suspicion of Wernicke's encephalopathy. Laboratory results were collected, and he was started on high-dose thiamine replacement. It turned out that the patient's whole-body thiamine levels were within normal range, indicating that he was not deficient and that this could not be the underlying cause. The next day, the patient was more alert and less confused but had continued bidirectional nystagmus and dysmetria due to his new-onset diplopia. The examination was improved when the left eye was covered. The patient's visual hallucinations persisted, so he was started on quetiapine along with melatonin to assist with sleep. Oral supplementation with magnesium chloride was continued. The next day, he no longer had visual hallucinations and had returned to baseline. His magnesium levels began stabilizing at 2.1 mg/dL for three consecutive days, and his dizziness subsequently decreased. CT of the chest, abdomen, and pelvis with and without contrast were ordered to rule out possible malignancy; all imaging was unremarkable. The patient continued to improve with the assistance of physical therapy and was shortly discharged from the hospital.

Outpatient follow-up one month after discharge showed a persistence of downbeat nystagmus. Repeat MRI once again did not show any intracranial processes. Per neurology, he was started on gabapentin 100 mg three times daily for the treatment of his nystagmus.

Three months prior to the current presentation, the patient had presented to the hospital with chronic diarrhea and right-sided chest pain. Further workup revealed a pulmonary embolism that was promptly and successfully treated over the hospital course. In regard to the chronic diarrhea, the patient had reported having 15 loose bowel movements per day for a week. During the time of that prior admission, the patient was also found to have a magnesium level of 0.3 mg/dL, which was treated promptly with 4 mg intravenous magnesium sulfate. A urine magnesium was ordered, which yielded a result of 1 mg/day, further indicating a gastrointestinal cause of magnesium loss over a renal cause. The patient was also found to have electrolyte abnormalities such as hypocalcemia (6.6 mg/dL), which was treated with 2 mg calcium gluconate infused over two hours. The patient also had hypokalemia at 2.7 mmol/L, which was treated with 40 mEq oral potassium chloride and 40 mEq intravenous potassium chloride. At time of discharge, the patient's magnesium level was 2.9 mg/dL, and all other electrolyte derangements had been resolved. Additional laboratory studies had shown low vitamin B12 levels, and he was provided oral replacement accordingly.

## Discussion

Due to recurring low magnesium levels in our patient as well as his severe neurological symptoms, we treated him in accordance with the current guidelines [8] for patients with severe symptomatic, hemodynamically stable hypomagnesemia. The patient was initially given 2 g of intravenous magnesium sulfate. Upon recheck, the patient's magnesium was still severely low, so he was given another load of 4 g of intravenous magnesium sulfate over a 12-hour period. Once the patient's magnesium levels returned to low-normal (1.8 mg/dL) levels, he was switched to oral magnesium supplementation. The patient was then given 64 mg sustained-release oral magnesium chloride twice daily. This was in conjunction with current treatment guidelines [8], which suggest that sustained-release oral magnesium chloride decreases renal and gastrointestinal losses associated with other formulations of magnesium supplementation (namely, magnesium oxide) [9].

According to current literature, hypomagnesemia is commonly caused by gastrointestinal losses, with the most common mechanism being chronic diarrhea [6]. Interestingly, in our case, three months prior to the current presentation, the patient had presented to the hospital with a secondary complaint of chronic diarrhea. During the time of that prior admission, the patient was also found to have a magnesium level of 0.3 mg/dL, which was treated promptly with 4 mg intravenous magnesium sulfate. The urine magnesium of 1 mg/day also indicated a gastrointestinal mechanism of magnesium loss. Three months later, at the time of the current presentation, the patient's magnesium level was found to be 0.4 mg/dL. However, a unique difference between the previous presentation was that the patient reported no diarrheal episodes in the three months leading up to this current presentation. In our literature review, we could not locate any case in which the patient had no recent history of diarrhea resulting in hypomagnesemia. While a diarrheal mechanism could be an explanation for this patient's past presentation of hypomagnesemia, the absence of any history of recent gastrointestinal symptoms at the time of the current presentation can rule out diarrhea as a possible cause of this patient's current hypomagnesemia.

In addition, the patient had become abstinent from alcohol after his last visit. In accordance with current literature, any attributable magnesium deficiencies should have been resolved in 2-3 weeks after his last drink (if alcoholism was the primary mechanism of magnesium losses) [3]. In the span of only three months, the magnesium levels in this patient had once again dropped to a severely low level despite no obvious gastrointestinal, renal, or alcohol-associated mechanism of electrolyte loss. Repeat vitamin B12 levels were found to be normal, indicating that the patient's nutritional status had not been completely compromised. However, upon further social history obtained from the patient's wife and sister, it was found that he had a remote history of diverticulitis first diagnosed in his early 30s. His father also had a history of diverticulitis from a young age as well. Although his most recent flare of diverticulitis was a couple of years prior to admission, the patient was susceptible to recurring bouts of diarrhea without an infectious etiology. He was scheduled for an outpatient colonoscopy to rule out any obvious cause of loose bowels. Additionally, an even more important factor was discovered indicating why this patient's magnesium levels dropped during this admission without diarrhea.

Due to the patient's altered mental status, a thorough history was obtained from the patient's family members, which revealed that the patient had COVID-19 five times over the last two and a half years. His most recent infection resulted in a significant intolerance to several foods. The patient's wife noticed that his diet had changed drastically as he would only eat oatmeal and jello. Both of the aforementioned foods have a combined less than 10%-15% daily value of magnesium. This provided additional supporting evidence as to why this patient was having recurring hypomagnesemia in the absence of diarrhea. The patient was given a nutritional consult inpatient and was also referred to an outpatient nutritionist to manage his care after discharge. The patient's recent dietary changes due to his COVID-induced taste changes coupled with his history of diverticulitis provide a logical explanation for his recurrent low magnesium levels resulting in severe neurological deficits. From the findings of our case, we wish to highlight the importance of obtaining a thorough history when evaluating patients with severe neurological symptoms as his social and medical histories were vital to providing direction in determining a clear diagnosis.

## Conclusions

This case exhibits the successful management of severe neurological symptoms due to low magnesium levels. Our case highlights the importance of considering hypomagnesemia higher on the differential when a patient presents with detrimental neurological deficiencies that are otherwise not identifiable by imaging or an endocrine, infectious, or autoimmune workup. We find this could be of particular importance in patient populations with a history of gastrointestinal diseases, as well as many who have developed taste issues following a COVID-19 infection. As this presentation is rare, it is important to first utilize first-line imaging and conduct a proper clinical workup before suspecting hypomagnesemia to be at the top of the differential diagnosis. With our case, we wish to highlight the importance of obtaining a thorough social and medical history in these patients, as it is crucial in determining an accurate holistic diagnosis.
